# A ResNet-50–UNet Hybrid with Whale Optimization Algorithm for Accurate Liver Tumor Segmentation

**DOI:** 10.3390/diagnostics15232975

**Published:** 2025-11-24

**Authors:** Proloy Kumar Mondol, Md Ariful Islam Mozumder, Hee Cheol Kim, Mohammad Hassan Ali Al-Onaizan, Dina S. M. Hassan, Mahmood Al-Bahri, Mohammed Saleh Ali Muthanna

**Affiliations:** 1 Institute of Digital Anti-Aging Healthcare, Inje University, Gimhae-si 50834, Republic of Korea; proloykumar1996@gmail.com (P.K.M.); arifulislamro@gmail.com (M.A.I.M.); heeki@inje.ac.kr (H.C.K.); 2 Next Station AI, Research & Development Center, Dhaka 1216, Bangladesh; 3 Department of Intelligent Systems Engineering, Faculty of Engineering and Design, Middle East University, Amman 11831, Jordan; m.alonaizan@meu.edu.jo; 4 Department of Information Technology, College of Computer and Information Sciences, Princess Nourah Bint Abdulrahman University, Riyadh P.O. Box 84428, Saudi Arabia; 5 Faculty of Computing and IT, Sohar University, Sohar 311, Oman; mbahri@su.edu.om (M.A.-B.); muthanna@sfedu.ru (M.S.A.M.); 6 Department of International Business Management, Tashkent State University of Economics, Tashkent 100066, Uzbekistan

**Keywords:** deep learning, bio-inspired optimization, ResNet-50, UNet, LiTs-UNet-WOA, Whale Optimization Algorithm, metaheuristic optimization

## Abstract

**Objective:** Segmentation of liver and liver tumors from 3D medical images is a challenging and computationally expensive task. Organs that are in close proximity may have similar shape, texture, and intensity, which makes it difficult for accurate segmentation. Accurate segmentation of liver tumors is important for diagnosis and treatment planning of liver cancer. **Methods:** A hybrid model with a U-Net based structure and the Whale Optimization Algorithm (WOA) was proposed. WOA was used to optimize the hyperparameters of the conventional LiTS-Res-UNet to obtain the best segmentation performance of the deep learning model. **Results:** The LiTS-Res-Unet + WOA hybrid model achieved a performance of 99.54% for accuracy, with a Dice coefficient of 92.38% and a Jaccard index of 86.73% on the benchmark dataset, outperforming state-of-the-art methods. **Conclusions:** The WOA-based adaptive search space was able to obtain an optimal set of hyperparameters for deep learning model convergence while increasing the accuracy of the model in the proposed hybrid model. The robust performance and clinical applicability of the model in liver tumor segmentation were demonstrated.

## 1. Introduction

The liver is a vital metabolic and digestive organ of the body that is the most common site of primary and metastatic tumors [1,2]. Liver tumors are the most frequent and lethal tumors in the world. Hepatocellular carcinoma (HCC) is one of the most common types of liver cancer. It is the third most common cause of cancer death, and the incidence of this type of cancer has been gradually increasing in the past few decades [2,3]. Dynamic medical imaging, especially computed tomography (CT) scans, has been extensively used in clinical practice for the diagnosis, staging, and treatment planning of HCC patients [4,5]. However, manually analyzing thousands of slices of CT images for each patient to find out the region of the tumor and to measure its size and extent can be an extremely time-consuming task even for the most expert radiologists, as it requires a high level of experience and expertise. Moreover, this can lead to fatigue and inefficiency in a longer analysis and potential misdiagnosis and can have serious impact on the physical and mental health of the physician [6]. Therefore, the use of automatic segmentation of HCC from CT medical images using Artificial Intelligence (AI)-based methods is critical to aid the clinicians. CT image-based Computer-Aided Diagnosis (CAD) systems can be extremely helpful in improving the diagnostic performance and decision-making capability of radiologists. Nowadays, deep learning algorithms such as convolutional neural networks (CNNs) that are capable of learning higher-level features are applied in the direct segmentation of liver tumors from raw medical images. Deep learning-based segmentation models have been popularly applied in the successful implementation of segmentation tasks, and their encoder–decoder design has produced the best performance for precise tumor delineation. Models like Fully Convolutional Network (FCN) and SegNet have helped to increase the accuracy of liver tumor classification [2,3]. The classification has been carried out using different machine learning based classifiers, such as Support Vector Machine (SVM), Random Forest (RF), and k-nearest neighbor (KNN), which differentiate between benign and malignant tumors. However, conventional methods often require expensive manual feature extraction, which inherently lacks the ability to capture the complex patterns of medical images. To address this issue, we can use transfer learning models such as CNN, VGG-16, and ResNet, which have been widely applied in segmentation and classification tasks. These models can directly process raw images and have the capability of extracting lesion features and classifying liver lesions as benign, malignant, or ambiguous. Even though the detection of lesions has achieved considerable progress, the classification performance of the models is somewhat limited by variations in tumor size, texture, and imaging modality as well as by class imbalance and the quality of images [3,4]. Stronger approaches that can bypass these shortcomings and provide more accurate and reliable segmentation and classification are required. In addition, an imbalanced class between benign and malignant tumors is a difficult problem. In most of the cancer data, the benign tumors are more abundant than the malignant ones. Therefore, the models are inclined to separate into a higher class and are unable to diagnose malignancy. Most of the segmentation algorithms like U-Net do not work well on low resolution or noisy images and are very costly. Therefore, more efficient, adaptive, and robust models are required to better detect and diagnose liver tumors [7]. Biologically motivated networks such as CNNs [5,8] have shown success in a variety of medical image analysis tasks such as organ segmentation [6], texture analysis [5], and disease classification [9]. Several research studies have reported that CNNs can provide good performance in cancer detection and diagnosis [10,11,12]. Nevertheless, the success of the segmentation and classification of the algorithms based on CNN models relies on the architecture and hyperparameters of the network that also impact on the performance of the CAD system. These parameters have a great impact on performance, and the algorithms based on natural phenomena can collaborate with deep learning models, exploring the solution space in a global way and finding the best hyperparameters. Conventional strategies for medical image processing have given way to innovative bio-inspired ones. Observed bio-inspired solutions are imitation of the behavior of animals, birds, and insects. Complex optimization problems are solved by such algorithms. The whale algorithm is a bio-inspired one of these algorithms and is based on the simulation of the behavior of the whale while attacking and hunting its prey. Particle Swarm Optimization (PSO) [13], Whale Optimization Algorithm (WOA) [14], Artificial Bee colony Optimization (ABC) [15], Differential Evolution (DE) [16], Harmony Search (HS) [17], Gravitational Search (GS) [18], Gray Wolf Optimization (GWO) [19], Antlion Optimization (ALO) [20], and Ant Colony Optimization (ACO) [21] are some well-known algorithms in this category. We implemented our study using WOA, as it can simulate the bubble-net hunting tactic deployed by humpback whales, which is suitable for maneuvering over the complex solution space of image segmentation that comprises multiple modes. Unlike PSO and other algorithms, which have the possibility of premature convergence or becoming stuck in local optima, WOA includes a linearly decreasing control parameter that adjusts the exploration strategy of the search from global to local search, which enhances the reliability of the convergence. In addition, the simplicity of the WOA structure, that contains fewer control parameters than the other algorithms, makes its implementation and the adjustment of parameters simple yet competitive (or superior) when compared to the other algorithms in terms of convergence speed, robustness, and segmentation accuracy. These benefits make WOA a good choice for image segmentation problems, where both accuracy and efficiency of computation are critical. Other research studies demonstrated that these algorithms were effective in solving complex optimization problems in medicine [22,23]. Unlike existing systems that are developed based on a feature engineering approach or a combined application of feature engineering and deep learning techniques, we propose a bio-inspired deep learning solution for liver cancer diagnosis with CT images. The hybridization of the combination of several deep learning models with WOA bio-inspired optimization was investigated for liver segmentation and feature extraction.

Even though the LiTs-Res-UNet architecture has attained promising outcomes, its performance is excessively reliant on human-selected hyperparameters, which might result in non-optimal convergence behavior. In this work, we propose a new hybrid LiTs-Res-UNet + WOA, in which the Whale Optimization Algorithm is applied for adaptively fine-tuning key hyperparameters for improved segmentation accuracy, robustness, and generalization. The contributions of this paper are as follows:

- We propose a novel hybrid optimization-segmentation framework called LiTs-Res-Unet + WOA, which integrates the Whale Optimization Algorithm (WOA) with a UNet-based architecture to automatically and adaptively tune the hyperparameters required for liver and tumor segmentation.
- Unlike prior studies that apply WOA to optimize a single parameter, we propose a closed-loop optimization framework in which WOA tunes a set of interdependent hyperparameters (learning rate, dropout rate, batch size) dynamically during training. This introduces a meta-optimization layer that is adaptive to the changing loss landscape of the ResNet-50–U-Net model.
- The inner level updates the network weights using backpropagation, and the outer level optimizes a population-based hyperparameter search using WOA by the validation Dice score. The convergence of validation performance shows that the hyperparameter optimization (HO) by the WOA outperforms the grid search and the Bayesian optimization methods because it achieves a better exploration–exploitation trade-off.
- Extensive experiments conducted on the LiTS17 benchmark dataset showed that the proposed model demonstrated state-of-the-art performance—with achieved pixel accuracy of 99.54%, Dice coefficient of 92.38%, and Jaccard index of 86.73%, respectively, which significantly outperformed existing segmentation techniques.
- The research results indicate that LiTs-UNet-WOA exhibits superior accuracy and robustness and has the potential to be an effective solution for liver tumor detection and treatment planning in real-life clinical applications.

The rest of the paper analyses the recent research and related issues in Section 2. Section 3 explains the methodology of the proposed work and the application of the optimized meta-heuristic approach. Section 4 presents the segmentation results. Section 5 and Section 6 represent the discussion and limitations of the proposed work, and Section 7 provides the conclusion of this work.

## 2. Related Work

**Figure 1:**
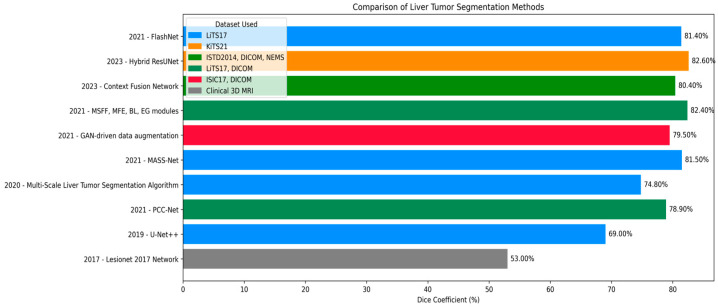
Dice coefficient comparison of liver tumor segmentation techniques across different datasets and publications.

Effective segmentation of liver tumors is one of the pivotal steps in medical image analysis, ranging from precise diagnosis and efficient treatment planning to follow-up monitoring of liver cancer. Many deep learning techniques have been developed to improve the accuracy and efficiency of liver tumor segmentation. In this review, we describe recent progress on segmentation algorithms and methods by highlighting the important works published in recent years based on different computational paradigms. An improved Artificial Bee Colony (IABC) algorithm and a general type-2 fuzzy possibilistic c-means (FPCM) were used in consistence with a CRF for brain tumor segmentation [24]. The method starts with removing annotations by a tracking algorithm and a given threshold. CRF estimates a conditional probability distribution based on image features to obtain posterior matrices for all image kernels. Then, the matrices are fed to the ABC algorithm for forming clusters, and the tumor cluster is determined from an adaptive threshold. It was evaluated on a sample of 100 MRI scans. Nevertheless, it depends on only one threshold calculated by ABC, which is suboptimal for liver segmentation because of the various lesion intensities. In [25], the segmentation of the whole liver in CT images is proposed based on the ABC algorithm as a clustering technique. The abdominal images were grouped into ABCs, and taking advantage of the sorted cluster centroids, extremum points were removed to establish the initial liver segmentation. This output was further refined by using a region-growing approach. This approach serves as a basis for the current study, which attempts to alleviate its weaknesses by using an alternative metaheuristic algorithm to obtain better performance. In [26], different transfer mechanisms and pheromone updating rules were implemented to work in the ACO framework for image segmentation, including edges, objects, and background. Fuzzy clustering was adopted to separate the background and foreground so that ants can work on boundaries effectively. Histogram peaks were used to determine the number of clusters and to initialize the clusters, optimizing efficiency and minimizing search time. But the method was evaluated on one image only and primarily focused on edge detection, thus being inapplicable to global liver segmentation. In [27], the Firefly Algorithm (FA) was employed in mammography image processing for breast tumor segmentation. Pattern matrix and kernel were used to measure pixel dissimilarity, generating a dissimilarity map. Subsequently, the labeling of suspicious regions was optimized by FA and refined by FCM clustering. Though effective for obviously discriminated cancerous tissues, this method is not competent for liver tissues with homogenous and inhomogeneous appearance, and it focuses on classification rather than full liver segmentation. An ABC-based approach was used in [28] to identify multiple image thresholds, yielding results equivalent to a near-exhaustive search. Its performance was compared with HBMO, PSO, Fast Otsu’s method, and the hybrid HCOCLPSO algorithm. Chen et al. [29] presented a Multi-Scale Liver Tumor Segmentation Network combining convolutional layers with Transformer-based architecture to handle short- and long-range dependencies. Evaluated on the LiTS17 dataset, it achieved Dice similarity coefficients of 0.920 for liver segmentation and 0.748 for tumor segmentation, improving baseline models while preserving fine-grained anatomical details. You et al. [30] proposed PGC-Net to perform contour-based parallel graph reasoning and the Pyramid Vision Transformer to extract multi-scale features. On LiTS17 and 3DIRCADb, it achieved mean Dice scores of 73.63% and 74.16%, but limitations remain in unclear tumor boundaries and inaccurate tumor margin delineation. He et al. [31] presented PAKS-Net, a 2D segmentation model leveraging global Swin Transformer features and a Position-Aware module to capture spatial relations of the tumor and adjacent organs. The model obtained tumor Dice scores of 0.893 (KiTS19), 0.769 (LiTS17), 0.598 (pancreas), and 0.738 (LOTUS), but still suffered from noise and slice-to-slice variability. Shui et al. [32] proposed a three-path neural network using MSFF, MFF, EI, and EG modules. On LiTS2017, it achieved a Dice coefficient of 85.55% and a Jaccard index of 81.11%. Cross-dataset validation reached Dice scores of 80.14% and 81.68% on 3DIRCADb and clinical datasets. Biswas et al. [33] introduced a GAN-based data augmentation method enhancing the quality and utility of training samples for biomedical image segmentation, yielding Dice scores of 0.908 on MIDAS, 0.872 on 3DIRCADb, and 0.605 on LiTS datasets. Wang et al. [34] proposed a Context Fusion Network using TSA modules and MSA skip connections, achieving Jaccard index of 81.56% and Dice score of 85.97% on LiTS2017, and 80.11% and 83.67% on 3DIRCADb. Muhammad et al. [35] introduced a hybrid ResUNet model combining ResNet and UNet for liver tumor segmentation on the MSD Task03 Liver dataset. The model, implemented in MONAI and PyTorch, reached Dice coefficients of 0.98 for tumor detection and 0.87 for segmentation. Fallahpoor et al. [36] used Isensee’s 2017 architecture for liver and lesion segmentation from 3D MRI, achieving mean Dice scores of 88% for liver and 53% for tumors on 128 patients’ T1w and T2w MRI images. Wang et al. [37] applied UNet++ for automated liver and tumor segmentation on MRI images of hepatocellular carcinoma patients, reaching a Dice score of 0.91 for liver and 0.612 for tumors. Recent advances (e.g., FasNet) indicate trends in medical imaging [38,39]. McGrath et al. [40] and Mojtahed et al. [41] illustrated the growing importance of semi-automated and deep learning methods. Wang et al. [42] and Vaidhya Venkadesh et al. [43] suggested incorporating multimodal imaging and historical patient records to improve performance [44]. Singh et al. [45] introduced FasNet, a deep learning model fusing ResNet-50 with VGG-16, incorporating Channel and Spatial Attention and Monte Carlo Dropout for enhanced liver tumor segmentation. Tested on LiTS17, FasNet achieved Dice of 0.8766 and Jaccard of 0.8487, outperforming several other methods in feature concentration, robustness, and uncertainty estimation. In [46], the authors demonstrated that GAN-generated samples can enhance segmentation performance, particularly for liver tumor imaging. They Achieved Dice scores of **0.908**, **0.872**, and **0.605** on the **MIDAS**, **3Dircadb**, and **LiTS** datasets, respectively, Data summaries are presented in Figure 1.

## 3. Methodology

**Figure 2:**
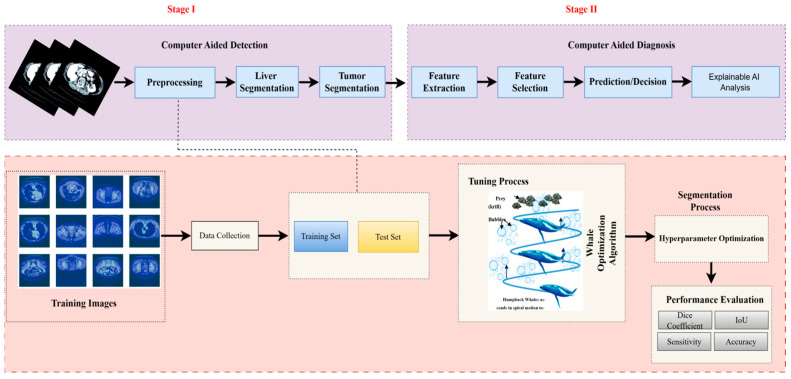
Overview of the proposed two-stage computer-aided liver tumor detection and diagnosis framework. Stage I (Computer-Aided Detection) involves preprocessing, liver and tumor segmentation, and feature extraction. Stage II (Computer-Aided Diagnosis) performs feature selection, prediction/decision making, and Explainable AI analysis. The Whale Optimization Algorithm (WOA) is employed for hyperparameter optimization, enhancing segmentation accuracy and performance metrics including Dice coefficient, IoU, sensitivity, and accuracy.

In this section, we outline the methods employed in our study. The pipeline of our proposed system is depicted in Figure 2. The first step is to construct the dataset, where three different views were obtained from the CT dataset. Subsequently, preprocessing is performed, followed by liver segmentation, and finally tumor segmentation. A summary of the materials and methods used in the current work, including the dataset, performance criteria, the state-of-the-art LiTs-Res-UNet, the bio-inspired novel architecture LiTs-Res-Unet + WOA with a metaheuristic algorithm, and the proposed framework, is presented in this section.

### 3.1. Dataset Description

We used publicly available standard data sets to validate and evaluate our liver tumor segmentation [47]. All experiments were performed on contrast-enhanced abdominal CT images from the LiTS2017 dataset (131 volumetric scans) and Radiopaedia (105 CT series). We did not use any MRI data in our experiments. Each volume is associated with a ground truth segmentation mask of the exact resolution that identifies the liver and, if present, hepatic tumors. These scans originate from different patient cases and scanning protocols, resulting in a mixture of anatomical presentations and imaging characteristics across the dataset. The second collection was extracted from Radiopaedia, a popular medical imaging database. It contains more than 105 liver CT series from many patients with a broad range of abdominal clinical presentations. While these scans are also in NIfTI format, the labeling quality and consistency vary in the same way as in real-world diagnostic settings. This dataset was added to improve the generalization performance and domain invariance of the model. All data were reconstructed into 2D axial slices. To ensure dataset consistency, each slice was resized to 128 × 128 × 3 pixels. CT slices were saved as JPEG files after applying suitable windowing (usually liver windowing with window = 150 and level = 30), and masks were saved as grayscale PNG files. This preprocessing step simplified storing and loading data on a standard PC with limited memory and facilitated integration with common deep-learning frameworks.

### 3.2. Dataset Preprocessing

**Figure 3:**
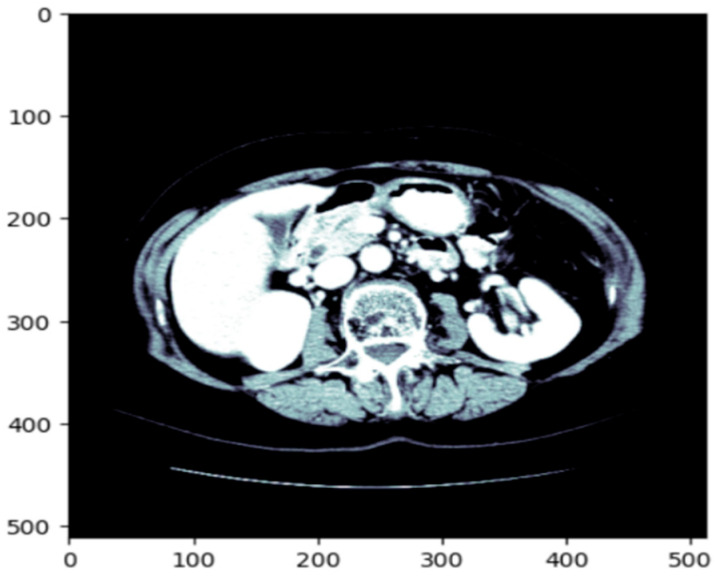
The original CT slice which enhances the contrast of the liver region to improve segmentation accuracy.

**Figure 4:**
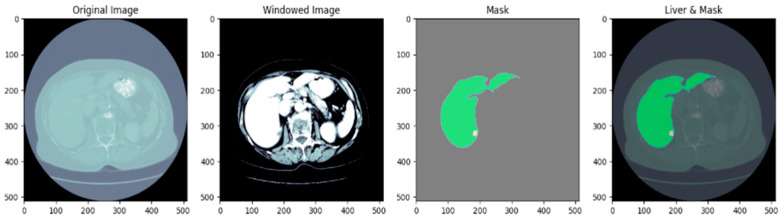
Preprocessing of CT scan: original image, windowed image, mask, liver, and mask overlay.

Before training the model, all volumetric CT images and corresponding masks were put through a structured preprocessing process. Initially, NIfTI (.nii) 3D volumes were loaded using the NiBabel library and reoriented to a standard axial orientation. Each volume was subsequently cut into individual 2D slices on the axial plane. Figure 3 shows a single axial slice of a CT scan after applying the windowing technique optimized for the liver. The windowing step improves the display of the liver and the surrounding tissue by adjusting the intensity range using the predetermined liver windowing parameters (width = 150, level = 30). The windowed bone colormap of an axial image can show the liver with respect to other structures in the abdomen by visually aiding segmentation. By adding the windowing, we can more clearly differentiate liver tissue from other organs, thus better supporting subsequent segments. Figure 4 depicts the unmodified input image (left), corresponding to the raw CT slice, and the windowed image (immediately left of the center), which improves the visibility of the liver by changing the range of intensities. The Mask (center-right) identifies the liver area, and the Liver and Mask Overlay (right) shows the liver area overlaid on the windowed image. This ensures more accurate segmentation and better visual perception. The slices were then resized to a fixed resolution of 128 × 128 × 3 pixels using bicubic interpolation for CT images and nearest-neighbor interpolation for masks to maintain categorical label correspondence. Additionally, histogram-based normalization and frequency-equalized scaling were optionally applied to enhance contrast uniformity among samples. CT images were saved in JPEG format for storage efficiency and compatibility with popular image processing pipelines, whereas binary masks were saved as PNG images containing a single grayscale channel.

**Figure 5:**
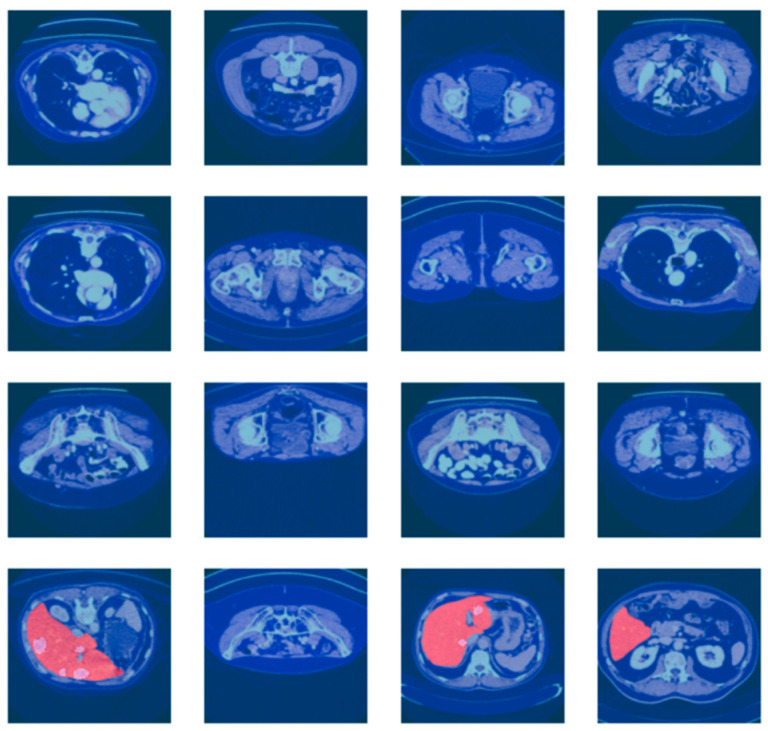
Batch of CT scan images with liver segmentation masks.

For more reliable experimental assessment, the merged dataset (LiTS17 + Radiopaedia) was split at the patient level (i.e., to prevent data leakage between splits) into train, validation, and test sets at a ratio of 70:10:20, ensuring that CT scans from the same patient were present in at most one of the splits. The large class imbalance between tumor and non-tumor regions was addressed by using a weighted loss function and balanced patch sampling at training time. While the current work reports results for a single fixed split, several training courses run with different random seeds were performed in the reported experiments to verify the stability of the results. These are the CT scan images with associated liver segmentation masks in Figure 5. Each of the images is preprocessed to focus on the liver region. The masks are used to indicate the location of the liver in the CT scan. The batch of images and masks shows the preprocessing and segmentation.

### 3.3. Based Model Architecture

#### 3.3.1. Pre-Trained Encoder

LiTs-Res-UNet is a deep encoder network, and ResNet-50 is adopted as the encoder to acquire high-quality contextual details. The ResNet-50 model, which has been pre-trained on multi-million ImageNet, contains fifty trained layers with residual blocks to reduce vanishing gradients. The point is that the error gradient propagated between the output and the input layers drops and is extremely small, which does not make it possible to learn in the initial layers. The residual block formula is the following:

(1) $$F\left(x\right)+x$$

**Figure 6:**
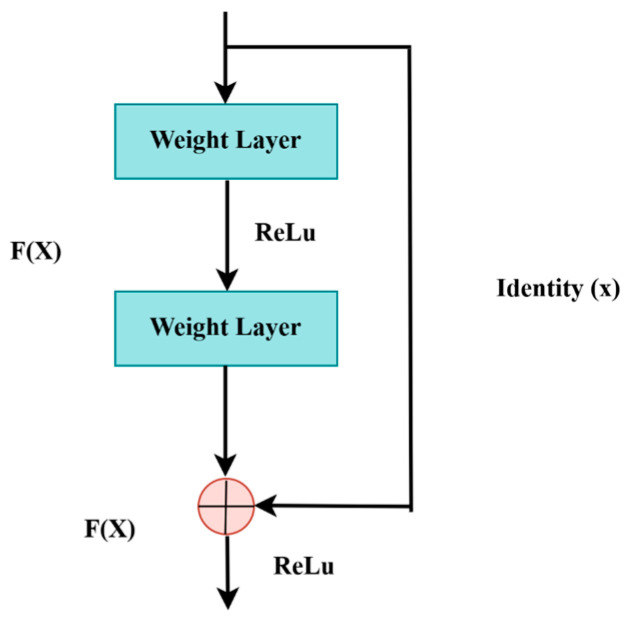
Residual block.

F(x) is the output of the layers and x is the input. The residual block is presented in Figure 6. The use of ResNet-50 as a feature extractor in reference to colon polyp segmentation is expected to improve the accuracy of the model.

#### 3.3.2. Model Decoder Path

**Figure 7:**
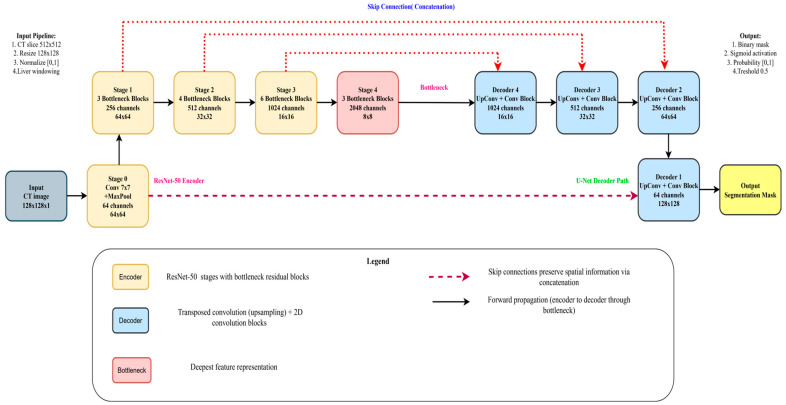
Proposed Hybrid LiTs-Res-UNet model architecture.

The LiTs-Res-UNet decoder progressively increases the resolution of the feature map by up-sampling to reassemble the spatial details lost during the encoding phase. These features are elaborated by the deeper convolutional layers in the sequence, and the batch normalization provides the stability of the learning process. Regularization methods, like dropout, reduce the problem of overfitting. Additional layers are transposed convolution (reverse convolution) layers, which increase the size of the feature map, increasing segmentation. Lastly, a 1 × 1 convolution with a sigmoid activation transforms the feature map into a probabilistic representation, saying whether each pixel is the object of interest or not, reconstructing the segmentation mask. The overall architecture of the LiTs-Res-UNet model is depicted in Figure 7.

### 3.4. Swarm-Based Whale Optimization Algorithm (WOA)

WOA [14] is a nature-based metaheuristic optimization method replicating the social model and hunting phase of humpback whales in nature, referred to as bubble-net hunting. It also falls into the swarm intelligence family of optimization algorithms inspired by the collective motion of flocks of birds or schools of fish, addressing problems. In this part, we discuss the Whale Optimization Algorithm’s theories, principles, and mathematical formulations.

#### 3.4.1. Motivation from Nature

Swarm Intelligence (SI) algorithms are based on the population and behavior of social animals, such as birds, fish, and mammals, and WOA is one such swarm-based technique. Despite large differences in their behavior as they move independently, these animals also show emergent collective behaviors that enable effective collaboration. Humpback whales, known for their smart and synchronized hunting techniques, are the primary models for WOA. One notable behavior is bubble-net feeding, in which a group of whale’s spirals upward through the water, releasing increasingly dense curtains of bubbles, driving fish or krill toward the surface and into their interior circle. This predation process is the main metaphor for the updating mechanism applied in WOA. In the optimization problem, to explore and exploit for enhancing the global optimum, the search agents (whales) operate in the search space. The optimization in WOA is performed using two primary steps: Exploration and Exploitation, which enable a trade-off between global search (searching new regions) and local search (refining solutions around the best solution found).

#### 3.4.2. Exploration and Exploitation Phases

WOA integrates two underlying mechanisms to mimic whale hunting behavior: the shrinking encircling mechanism and the spiral movement.

- **Exploration:** This process iteratively traverses the search dimensions in search of the global optimum. During this phase, whales move randomly to search for unvisited regions. The search is guided by the position of the best solution found so far, allowing the algorithm to escape local optima.
- **Exploitation**: This phase focuses on refining the search around the best solution detected. Whales use the shrinking (enclosing) mechanism along with the spiral path, updating their position by considering the spiral trajectory, which converges more effectively to the global optimum.

**Figure 8:**
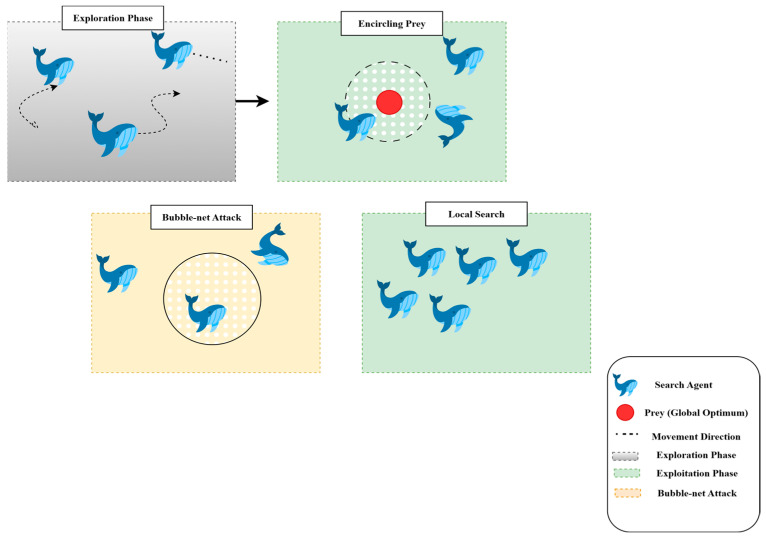
Hunting strategies of the whale optimization algorithm.

These two stages, shown in Figure 8, cooperate to allow WOA to find a trade-off between diversification (global exploration) and intensification (local exploitation).

### 3.5. Proposed U-Net Segmentation with WOA

Here, we present the U-Net segmentation model to liver tumor segmentation, and hyperparameter optimization of the model (learning rate, dropout rate, and batch size) using the Whale Optimization Algorithm (WOA). First, we define the U-Net Segmentation Problem class, inheriting the Problem class from the Mealpy library. This class wraps up the optimization problem by defining an objective function, which accepts a candidate solution (hyperparameters set) and decodes this solution to the learning rate, dropout rate, and batch size. These hyperparameters are subsequently employed to construct and compile a U-Net model and to fit it for a given number of epochs on the given training sets (train images and train masks) and to predict on the given test sets (test images and test masks). The objective function is the test accuracy to be maximized for optimization.

The U-Net model directly forms an encoder–decoder network. The features of the input image are extracted by the encoder through convolutional layers, with dropout layers to prevent overfitting. The feature maps are sampled, and convolutions are applied in the decoder to predict the segmentation mask. The output of the model is resized using a Lambda layer at the expected 128 × 128 pixel size. The output is passed through a sigmoid to obtain a final binary segmentation mask.

The Whale Optimization Algorithm (WOA) is used to do hyperparameter tuning. WOA is an evolutionary-based optimization method proposed by Mirjalili [14] through an observation of the hunting style of humpback whales that keeps the tradeoff between the explorative and exploitative style. It works by cycling through a population of potential solutions (whales) and moving its position with the best solution encountered up to that point. This makes Anytime Net capable of effectively finding the optimum number for learning rate, dropout rate, and batch size. Boundaries for these hyperparameters are specified in the code, and the optimization is performed for a certain number of iterations. The model with the best solution is chosen, using the test set accuracy to guide the decision.

**Figure 9:**
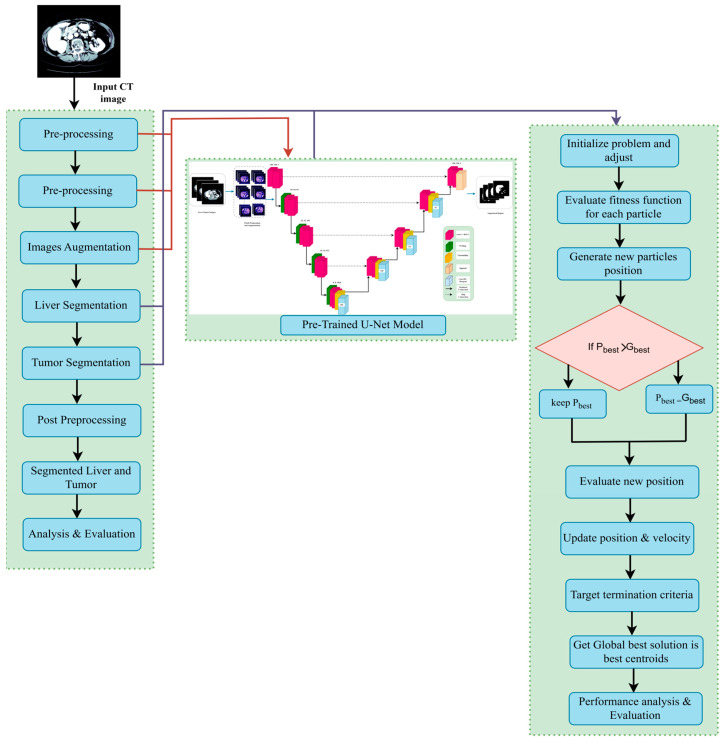
The proposed LiTs-Res-Unet + WOA approach.

Once the optimization process has been finished, the best agent (hyperparameter set) is found and the corresponding accuracy and optimized hyperparameters (learning rate, dropout and batch size) are printed. This framework is successful to couple deep learning with nature solved optimization approaches in order to improve the performance of the segmentation model, and it becomes a good choice for liver tumor segmentation. As demonstrated in Algorithm 1, the optimal hyperparameters for liver segmentation are computed. The overview of the proposed LiTs-Res-Unet + WOA optimized clustering with bio-inspired WOA for enhanced liver tumor CT image segmentation is shown in Figure 9.

To better visualize the interaction between WOA and the gradient-based learning procedure in ResNet-50–UNet, we assume that each whale in the WOA population corresponds to a candidate vector H = [learning rate, dropout rate, batch size] in hyperparameter space. For each candidate H, the ResNet-50–UNet network is trained using the regular backpropagation-based training for a few epochs, and the achieved validation Dice score is recorded as the fitness value. Then, the corresponding hyperparameter vectors are updated using WOA’s exploration (global search) and exploitation (local refinement) phases. During exploration, the whales perform random walks in the hyperparameter search space to find new candidate vectors, and, during exploitation, the whales update their position vectors using the shrinking and spiral procedures in WOA to steer the population towards the best candidate found so far. The adaptive process continues until convergence, enabling the model to automatically find an optimal set of hyperparameters that provides better segmentation accuracy and convergence than a manually tuned model. While the ResNet-50–UNet’s internal training procedure using gradients does not change, WOA is used externally as a hyperparameter-guided meta-optimizer.

**Algorithm 1:** Proposed WOA-driven meta-optimization framework integrated with ResNet-50–UNet for adaptive liver tumor segmentation.**Input:**       -Training images: X_train, masks Y_train       -Validation images: X_val, masks Y_val       -WOA parameters: N (population size), T_max (max iterations)       -Hyperparameter bounds: Ω = [η_min, η_max] × [*ρ*_min, *ρ*_max] × [β_min, β_max]**Output:**       -Optimal hyperparameters H* = [η*, ρ*, β*]        -Trained segmentation model M*
1: Initialize **N whales** with random positions H_i ∈ Ω, i = 1,……,N 2: Set a = 2 (WOA control parameter) 3: **for** t = 1 to T_max **do**
4:        **for each whale** i = 1 to N **do**
5:              M_i ← **Train_ResNet50_UNet** (X_train, Y_train, H_i, epochs = 5)
6:              Dice_i ← **Evaluate_Dice** (M_i, X_val, Y_val)
7:            Stability_i ← 1 − std(val_loss_last_10_epochs)
8:            F_i ← 0.7 × Dice_i + 0.3 × Stability_i
9:        **end for**
10:        H* ← argmax_{H_i} F_i (Identify best solution)
11:        **for each whale** i = 1 to N **do**
12:              Update **a, A, C, l, p**
13:              **if** *p* < 0.5 **then**
14:                   **if** |A| < 1 **then** (Exploitation)
15:                           D ← |C · H* − H_i|
16:                           H_i ← H* − A · D
17:                   **else**
18:                           H_rand ← Random whale position (Exploitation)
19:                           D ← |C · H_rand − H_i|
20:                           H_i ← H_rand − A · D
21:                   **end if**
22:            **else**
23:                           // Spiral update (exploitation)
24:                           D′ ← |H* − H_i|
25:                           H_i ← D′ · exp(b·l) · cos(2πl) + H*
26:                   **end if**
27:                   // Ensure bounds
28:                   H_i ← Clip(H_i, Ω)
29:           **end for**
30:           // Linear decrease of a from 2 to 0
31:           a ← 2 − 2·t/T_max
32: end for
33: M* ← **Train_ResNet50_UNet** (X_train, Y_train, H*, epochs = 100)
34: **return** H*, M*

#### 3.5.1. Hyperparameter Tuning with Whale Optimization Algorithm

In this study, the hyperparameters for the Whale Optimization Algorithm are set to ensure effective training of the model. These parameters, especially the weights assigned to each loss function, enable precise tuning, which leads to improved performance in liver tumor segmentation. In Table 1, WOA is employed to optimize these hyperparameters, aiming to achieve high accuracy and robustness in identifying liver tumors from medical imaging data.

#### 3.5.2. Mathematical Formulation of WOA-Segmentation Coupling

A bi-level optimization framework is formed by combining the Whale Optimization Algorithm with ResNet-50–U-Net, which helps solve the problem of hyperparameter tuning for medical image segmentation. This is because conventional methods are based on single-level optimization, where the weights of the network are determined by gradient descent, while our proposed method adds an additional outer optimization loop that adaptively searches the hyperparameter space and an inner loop to perform normal backpropagation training.

- **Problem Formulation:**

Let H=[η,ρ,β] denote the hyperparameter vector consisting of learning rate (η), dropout rate (ρ), and batch size (β). The hyperparameter space is bounded by Ω = [η_min, η_max] × [ρ_min, ρ_max] × [β_min, β_max], where the bounds are defined in Table 1. The optimization objective is formulated as follows:

$$H^{*}=arg\;\underset{H\in \varphi }{\max}F(H)$$

where the fitness function F(H) evaluates the segmentation quality achieved by the ResNet-50–U-Net model when trained with hyperparameters H.

- **Fitness Function Design:**

The fitness function combines segmentation accuracy with convergence stability:

F(H)=α⋅Dice(H)+(1−α)⋅Stability(H)

where Dice (H): Validation Dice coefficient obtained after training the model with H for E epochs, Stability(H) = 1 − σ(L_val): Convergence stability measured by the standard deviation of validation loss over the final 10 epochs, α = 0.7: Weighting parameter that prioritizes segmentation accuracy while ensuring stable convergence.

This multi-objective formulation prevents the optimizer from converging to hyperparameter sets that achieve high validation scores through overfitting or unstable training dynamics.

- **Segmentation Loss Function:**

For a given hyperparameter set H, the ResNet-50–U-Net model is trained using a composite loss function:

$$L_{\mathrm{seg}}(W;H)=-\mathrm{Dice}(\hat{Y},Y)+\lambda_{1}\cdot L_{\mathrm{BCE}}(\hat{Y},Y)+\lambda_{2}\cdot L_{\mathrm{Focal}}(\hat{Y},Y)$$

where W is network weights (learned via gradient descent), $\hat{Y}$ Predicted segmentation mask, Y is Ground truth mask, L_BCE: Binary cross-entropy loss, L_Focal is focal loss with γ = 2 to address class imbalance, λ_1_ = 0.5, λ_2_ = 0.3: Loss component weights, and the Dice loss component is defined as follows:

$$\mathrm{Dice}(\hat{Y},Y)=\frac{2\sum_{i=1}^{N}{\hat{y}}_{i}y_{i}+\epsilon }{\sum_{i=1}^{N}{\hat{y}}_{i}+\sum_{i=1}^{N}y_{i}+\epsilon }$$

where N is the number of pixels, and ε = 1 is a smoothing constant to prevent division by zero.

- **WOA-Driven Hyperparameter Update:**

In the WOA framework, each whale i corresponds to a candidate hyperparameter solution H_i (t) in iteration t. The total population of N whales is used to search in the hyperparameter space Ω. The way in which a whale updates its position is divided into two behavioral modes:

**(1) Encircling Prey (Exploitation):** When a promising hyperparameter region is identified (|A| < 1 and *p* < 0.5), whales converge toward the best solution H*(t):

$$\begin{array}{l} D=|C\cdot H^{*}(t)-H_{i}(t)|\\ H_{i}(t+1)=H^{*}(t)-A\cdot D \end{array}$$

where C = 2r is random vector with r ∈ [0, 1] that introduces stochastic perturbations.

A = 2a · r − a: Coefficient vector that controls the step size, a: Linear parameter decreasing from 2 to 0 over iterations: a = 2(1 − t/T_max).

**(2) Spiral Update (Exploitation):** To mimic the spiral swimming pattern of humpback whales (*p* ≥ 0.5), positions are updated using the following:

$$\begin{array}{l} D^{\prime }=|H^{*}(t)-H_{i}(t)|\\ H_{i}(t+1)=D^{\prime }\cdot e^{bl}\cdot cos(2\pi l)+H^{*}(t) \end{array}$$

where b = 1: Constant defining the logarithmic spiral shape, and l is a random number in [−1, 1] controlling the distance from the spiral center.

**(3) Search for Prey (Exploration):** When |A| ≥ 1 and *p* < 0.5, whales explore distant regions by moving toward randomly selected positions:

$$D=|C\cdot H_{\mathrm{rand}}(t)-H_{i}(t)|\;H_{i}(t+1)=H_{\mathrm{rand}}(t)-A\cdot D$$

where H_rand(t) is a randomly selected whale position from the current population.

- **Boundary Enforcement:**

After each update, hyperparameter values are clipped to ensure feasibility:

$$H_{i}(t+1)=\mathrm{Clip}(H_{i}(t+1),\Omega )$$

This prevents the optimizer from exploring physically meaningless regions (e.g., negative learning rates or batch sizes exceeding memory capacity).

- **Convergence Criterion:**

The optimization terminates when either of the following occur:

The maximum iteration count T_max = 100 is reached, or The fitness improvement falls below threshold ε_conv = 10^−4^ for 5 consecutive iterations:

$$|F(H^{*}(t))-F(H^{*}(t-1))|<\epsilon_{\mathrm{conv}}$$

### 3.6. ResNet-50 + U-Net

Multi-Level ResNet-50 with bottleneck aggregation of the WOA algorithm aim to extract and aggregate deep features and comprise the framework of segmentation. The hierarchical structure is used, and the combination of ResNet-50 blocks enables multi-scale convolutional representation, whereas bottleneck layers are used that enable the reduction of computational expense without sacrificing vital information.

**Step 1:** Feature Extraction ResNet-50 blocks, containing different receptive fields (e.g., 1 × 1, 3 × 3, 5 × 5) as well as residual learning, processing the input image to facilitate rich spatial encoding and gradient stability. A bottleneck layer follows each block, which entails compressing the features maps and decreasing the channel number, increasing efficiency.

**Step 2:** Hierarchical Aggregation. The outputs of bottlenecks are hierarchically integrated by over skipping. Feature maps of higher levels are up sampled and put together with lower-level ones to make the possibility to apply deep skip fusion between the levels. This advances improvement in semantic context fusion and localization—an important requirement in medical image segmentation.

**Step 3:** Final Merging and Production. Each of the aggregated features is concatenated and sent to a set of convolutional layers to result in a refined feature map. This map engulfs the context of the world and the structure of the region, and the segmentation is great.

#### 3.6.1. Encoder

**Figure 10:**
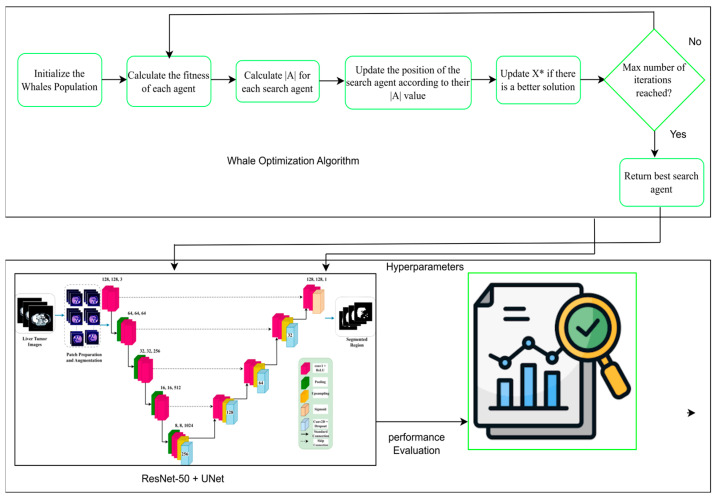
Overall architecture of the proposed WOA for liver and tumor segmentation. This X* means that the best solution is found during the optimization process.

As Figure 10 shows, the encoder component of the U-Net + ResNet-50 model plays a key role in extracting the feature of the input image. The encoder consists of several convolutional layers whose sizes transform the spatial dimensions of the input to smaller sizes and the number of feature maps to higher. This paper takes advantage of the unique U-Net architecture, where the skip connections are used to save the spatial information and underlines its suitability for medical image segmentation. In the normal U-Net architecture, the encoder comprises a few convolutional blocks combined with down-sampling layers. The network also learns various abstraction abilities since there is a multi-convolutional layer within every block. In general, pooling layers (most commonly max pooling) are applied to down-sample the size of feature maps to make the model sensitive to the global structural attributes at a trade-off of local detail. A deep encoder containing ResNet-50 modules improves multi-scale feature extraction ability. The Inception module combines parallel convolutional branches using kernel sizes, thus learning an effective spatial hierarchy. The given method can be especially useful to cover a tumor’s size and shape variation, which one often faces during medical imaging. The convolutional operations provide a global view of the features representation through which the segmentation tasks on tumors are performed with better results. In addition, the encoder positively impacts the efficiency of the segmentation process. A feature swapping structure is also integrated, allowing the feature maps to be compressed before passing them to the subsequent layers, tailoring the encoder to achieve an optimal, balanced trade-off between efficiency and accuracy. Such an architectural solution enables the model and the large size of medical image sets without compromising the quality of the resulting segmentation results, which would apply in clinical use. In short, the encoder of the proposed U-Net + ResNet-50 structure provides a strong and healthy basis in feature extraction, which prepares the informative feature to feed the decoder and segmentation process. The design of the proposed WOA module follows a multi-level encoder–decoder strategy, where modules of the ResNet-50 backbone are deployed at each scale. With some previous connection of stamp bypass, such modules ensure that the context and spatial information are not lost between layers. The down-sampling is performed on input size 128 × 128 images over four encoder stages: 64 × 64, 32 × 32, 16 × 16, 8 × 8. In each step, ResNet-50 modules are applied to obtain local-global feature representations. The features are then pooled into a bottleneck representation before decoding along a symmetric up-sampling path. The decoder path involves skipping connections to the opposite encoder levels and the historical stamp modules, allowing fine-grained details to be recovered. The output is then unraveled to its initial dimension (128 × 128) using a chain of convolution and up-sample layers. The high-order modules used throughout the pipeline are defined in the legend on the bottom right of the figure. This architecture reduces the error of segmentation and error delamination of boundaries and enacts multi-level feature aggregation to guarantee spatial consistencies.

#### 3.6.2. Decoder

The task of the U-Net + ResNet-50 design decoder is to reassemble the encoded feature representations into the final segmentation mask. Its major goal is the gradual reconstruction and alignments of the downsized feature maps produced by the encoder to the initial input spatial resolution. To tackle this, the decoder utilizes a set of transposed convolutional layers (commonly known as deconvolutions) or convolution procedure and up-sampling layers. Structurally, the decoder is the same as the encoder with reversal of the direction and a series of operations with every up-sampling step doubling the size of the feature maps in spatial relationship. Skip connections with the respective encoder layers are added so that the decoder can have access to high-resolution features that were down sampled during the encoding processes. The linking structures maintain important spatial information that is necessary in the precise location of the tumor boundary in the medical images. Another step taken to mitigate the shortcomings is by adding Inception modules in the decoder to foster greater learning of multi-scale features to refine the segmentation output. Unlike the encoder, ResNet-50 modules that are inbuilt in the decoder support capturing and decoding multiple structure representations of hierarchies that are at various scales. The capability to reconstruct fine-grained details and, simultaneously, stay aware of the wider information in the image, enabled by this dual capability in particular, can be particularly useful when it comes to the detection of irregularly shaped liver tumors. Further, the decoder is also required to reconcile segmentation accuracy and computational efficiency. This trade-off between complexity of the model and overall efficiency can be negotiated well by a careful choice of the number of feature maps to be deployed at each layer. Finally, the decoder within the U-Net + ResNet-50 architecture has a decisive role in enhancing feature representations, which perform a significant role in the generation of high-quality segmentation masks with clinically sound performance.

#### 3.6.3. Bottleneck

The bottleneck in the U-net + ResNet-50 model denotes the point of maximal feature dimensionality between the encoder and the decoder. The stage is important especially because it focuses on the most pertinent concepts of the input data and cuts on less important information. The bottleneck architecture can thereby increase the overall performance of the model in carrying out complicated tasks like the segmentation of liver tumors. In the U-Net + ResNet-50 system, the bottleneck retains only a small number of feature maps by combining Inception modules, guaranteeing that important features are maintained. The 1 × 1 convolutions of these modules allow it to create significant compressions with little information loss as a threat. In addition to dealing with the resource limitations, this dimensionality reduction also empowers the model to obtain generalization of previously unseen data. The bottleneck is such a place where features retrieved at several abstraction levels are integrated. The tradeoff between computational complexity and performance is created by the design itself. When examined architecturally, one tends to think of two possible paths: scale the number of Inception modules or scale the number of feature maps per module. Its goal is to keep the bottleneck appropriately restrictive and to keep the network, thus, the power of its representations. Finally, it is the bottleneck structure in the proposed U-Net + ResNet-50 architecture that is of central importance to achieve better segmentation accuracy especially in the detection of liver tumors.

#### 3.6.4. ResNet-50 Backbone

The backbone of U-Net, which in this architecture is ResNet-50, constitutes the main feature extractor and directly impacts the segmentation quality [48]. The backbone plays the role of the structural base network, in which the first features are obtained before the other functionalities of the U-Net model are carried out. Backbone choice is also important to establish whether the model can work in many imaging modalities, which is important, especially in the medical community, where segmentation of tumors of the liver is important. The ResNet-50 is particularly good as a backbone due to the multiple parallel convolutional paths exploring contrasting kernel sizes in the network. This design will allow the network to extract a wide collection of feature representations and be better positioned to realize the structure size and position variations. Meanwhile, the backbone should not have excessive computational overhead. Using transfer learning, our U-Net + ResNet-50 model is more likely to converge to higher accuracy in fewer training epochs using pre-trained weights on large-scale datasets. This property’s usefulness is particularly high in medical imaging applications where labeled datasets are sparse. Nonetheless, not all U-Net-based frameworks would take optimal advantage of using hierarchical feature representation, which Inception modules offer. This drawback may restrict the model from detecting complicated relationships between parts of an image. This capability is essential where the different types of liver tumors may have lesions of identical morphological similarities. The suggested U-Net + ResNet-50 rides this by incorporating Inception modules, thus it presents a good backbone that can support high performance and advanced clinical applicability. The support of Inception modules alleviates several general issues in medical picture separation. This ability to obtain multi-scale is particularly beneficial, as tumors occur in various shapes and sizes. No one size of kernel can be expected to work well with this diversity. The ResNet-50 backbone can analyze tumor features on many scales, as each branch has convolutional layers enacted in parallel with kernels of varying sizes. Also, 1 × 1 convolutions in the Inception modules perform dimensionality reduction. It minimizes parameters with necessary information being maintained—a property that is extremely useful in medical imaging terms and scenarios where computational facilities are not always available. This reduces the computational requirements by optimizing pattern recognition with no performance loss and facilitates the applications that demand real-time clinical deployment, such as ResNet-50. Moreover, the Inception modules improve feature combination by combining the information across the convolutional branches, which makes the abstract representation of tumor features stronger. Such a mechanism enhances the capability of acquiring dense details on liver tumors, which results in greater segmentation accuracy. The hierarchical representation of features that the Inception design offers further enhances the information flow in the model, allowing more detailed outputs and further improvement in segmentation quality overall.

### 3.7. Explainable AI (XAI)

Explainable AI (XAI) [49] on the other hand is prevalent in medical image segmentation, where the credibility of a model and its transparency are crucial in medical informatics shown in Figure 10. Two commonly used methods of achieving interpretability in image segmentation tasks are gradient-weighted Class Activation Mapping (Grad-CAM) and LIME (Local Interpretable Model-agnostic Explanations). Grad-CAM, a local interpretation, computes gradients of the target class with respect to the ultimate convolutional layer feature maps, and generates a heatmap, which indicates the regions of an image that contribute the most to a segmentation choice. The Grad-CAM model at pixel A_k_ represents a class score y as follows:

$$Grad\text{\_}CAM=ReLU(\sum_{k}\alpha_{k}.A_{k})$$

where $\alpha_{k}$ is the weight of each feature map $A_{k}$, and the ReLU function guarantees that only positive contributions are considered, indicating the informative regions for segmentation.

SHAP provides post hoc explanations to the predictions of a model by attributing an importance to each feature (e.g., pixel or region) that contributes to the model’s decision. For segmentation, SHAP highlights assist in pinpointing where in an image is positioned the strongest effect on segmentation decision for tumor and non-tumor pixels, and thus establishes clinical trust and model transparency. SHAP is based on cooperative game theory and computes the Shapley value for each input feature. The value for feature $\varphi_{i}$ in the Shapley value is defined as below:

$$\varphi_{i}=\sum_{S\subseteq N\setminus \{i\}}\frac{|S|!(|N|-|S|-1)!}{\left|N\right|!}[f(S\cup \{i\})-f(S)]$$

where N is the number of input features, S is a sub-set of N with the absence of feature i, f(S) is the value of the model’s prediction when using only the features that are related to S. In the case of image segmentation, f(S) is the model’s predicted probability that a pixel is in the tumor class based on only the features in subset S. SHAP values therefore measure the incremental value of each pixel or region tor the final prediction.

**Figure 11:**
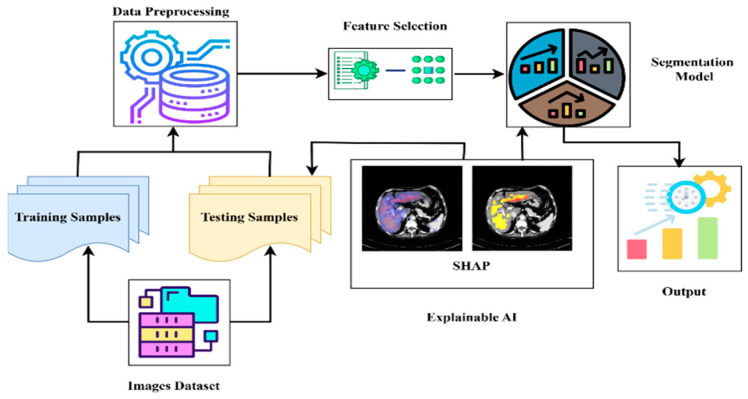
Explainable AI analysis.

In this study, we used SHAP and Grad-CAM because they provide a more accurate pixel-level explanation, which is what we need for segmentation. In contrast to Grad-CAM’s rough heatmaps, SHAP consistently provides (theoretically motivated) explanations of how much each pixel affects the model’s prediction. Figure 11 shows explainable AI analysis. 

### 3.8. Performance Measures

This paper was used to verify the correctness of liver lesion segmentation by the proposed hybrid algorithm and to compare it with the state-of-the-art algorithm based on three indices: the Jaccard index [50], Dice coefficient [51], and correlation coefficient [52].

**Jaccard index:** This is the similar index of binary data below where AOO is the overlapping area, M is a binary image, and K is a ground truth image:

$$J\left(AOO\right)=\frac{M\cap K}{M\cup K}$$

**Dice Index:** This is a co-efficient to compute segmentation. The Dice coefficient value is the fraction of the predicted image pixels that align with the ground truth pixel-wise:

$$D\left(M,K\right)=\frac{2|M\cap K|}{\left|M\right|+|K|}$$

**Accuracy:** Accuracy is the likelihood of hit prediction, and it can be determined:

$$\mathrm{Accuracy}=\frac{True\;Positive\;+\;True\;Negative}{True\;Positive\;+\;True\;Negative\;+\;False\;Positive\;+\;False\;Negative}\;$$

## 4. Experimental Results and Discussions

### 4.1. Implementation

The architecture was deployed with PyTorch 2.1.1. All the training and experimental tests were carried out on a personal workstation using the NVIDIA GeForce RTX 3090 (NVIDIA, Santa Clara, CA, USA) and the Intel(R) Core (TM) i7-10700KF CPU 3.80 GHz (32 GB) (Taiwan Semiconductor Manufacturing Company (TSMC), Hsinchu, Taiwan). Some other segmentation models were used as a comparison, such as Swin Transformer and the EfficientNetB0-UNet. CT images were resized from 512 × 512 to 128 × 128 pixels, intensity normalized to the [0, 1] range, and underwent liver-specific windowing. Random rotation, flip, and intensity shift were used for data augmentation. We used PyTorch 2.1 with MONAI 1.1 and standard scientific Python libraries (NumPy 1.25, SciPy 1.11). The Whale Optimization Algorithm (WOA) was used for hyperparameter optimization. Hyperparameter optimization was conducted using a population size of 20 and the number of epochs = 100.

On the computational side, WOA-based optimization converged in five outer iterations, with a population size of 20 whales. This resulted in 14 percent less total training epochs before the validation accuracy began to stabilize in comparison to the hyperparameter values selected by hand with either Adam. The marginal training overhead introduced by the WOA was negligible since it is only used during the hyperparameter search and does not impact the prediction/inference time. We report an average per-slice inference latency of 14.3 (ms) for a single 2D CT slice on an RTX 3090 GPU, which further validates that the proposed WOA integration speeds up the training process but does not increase the prediction time. The overall complexity of the algorithm is in the order of the number of whales times the number of hyperparameters, which is low.

### 4.2. Ablation Study

We performed a detailed ablation experiment to further verify the role of each part of our LiTs-Res-UNet + WOA architecture. Table 2 shows comparative analysis of the segmentation results of the proposed four model variants is presented. The baseline U-Net model demonstrated relatively moderate accuracy (Dice: 78.45%, Jaccard: 64.67%). The inclusion of WOA to enhance model hyperparameters resulted in improved performance in terms of Dice (82.13%) and Jaccard (69.85%), confirming the effectiveness of the metaheuristic optimization algorithm. When applying the ResNet-50 backbone, the results continued to increase (Dice: 87.66%, Jaccard: 78.24%). However, it is important to note that this improvement was accompanied by a significant increase in model complexity and training time. In comparison, the proposed LiTs-Res-UNet + WOA model achieved the highest level of accuracy, with Dice: 92.38%, Jaccard: 86.73%, and Pixel Accuracy: 99.54%, while maintaining an acceptable and efficient training time, which confirms the potential of combining the above-mentioned techniques for liver tumor segmentation.

### 4.3. Statistical Significance Analysis

We measured the relative contribution of architectural changes and hyperparameter optimization, and we conducted paired *t*-tests for each model variant against the baseline in Table 3. Contributions of WOA Optimization (U-Net vs. U-Net + WOA): After integrating WOA, Dice increased by +3.68% (*p* = 0.003) and Jaccard by +5.18% (*p* = 0.002), and training time decreased by 13.4%, suggesting that WOA optimization alone leads to notable performance gains and improved training efficiency. Contributions of ResNet-50 Encoder (U-Net vs. ResNet-50 U-Net): Replacing the default encoder with ResNet-50 resulted in significant segmentation quality improvements: Dice increased by +9.21% (*p* < 0.001) and Jaccard by +13.57% (*p* < 0.001), demonstrating the benefits of deeper feature extraction. Additive Effect (ResNet-50 U-Net vs. LiTs-Res-UNet + WOA): Hyperparameter tuning of ResNet-50 U-Net with WOA led to additional improvements of +4.72% in Dice (*p* = 0.001) and +8.49% in Jaccard (*p* < 0.001), along with a 13.6% reduction in training time compared to manual parameter tuning. This demonstrates that the combination of advanced architecture and metaheuristic optimization results in statistically significant gains beyond the individual components.

### 4.4. Comparison

Table 4 reports the results of some of the latest algorithms in liver tumor segmentation. Classical multi-scale methods [30] and PGC-Net [31] record Dice scores of 74.8 and 73.63, respectively, whereas PAKS-Net [34] enhances the performance to 76.9. The introduction of sophisticated algorithms like MSFF, MFF, EI, and EG improves the accuracy of segment outputs to 85.55 Dice and 81.11 Jaccard [33]. Context Fusion Networks in Twin-Split Attention and MSA skip connections [35] perform the same with 85.97% Dice and 81.56% Jaccard, and a hybrid framework, FasNet [45], accomplishes 87.66% Dice and 84.87% Jaccard. LiTs-Res-UNet shows 79.25 Dice and 65.88 Jaccard is competitive as a baseline result. Optimized with the Whale Optimization Algorithm (WOA), the model achieves 92.38% Dice and 86.73% Jaccard, which, in turn, are far better than those of the existing state-of-the-art methods. Notably, these findings confirm the effective combination of deep residual learning with metaheuristic optimization in liver tumor segmentation.

**Figure 12:**
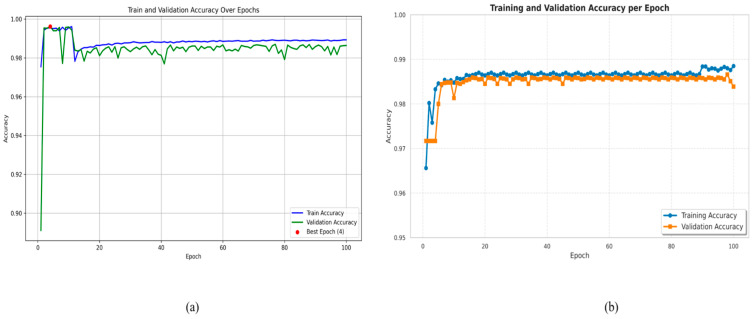
Training and validation accuracy over epochs. (**a**) LiTs-Res-UNet; (**b**) LiTs-Res-UNet + WOA.

Training and validation accuracy versus epochs of Figure 12a LiTs-Res-UNet that includes and does not include the Whale Optimization Algorithm (WOA). The optimized Figure 12b LiTs-Res-UNet + WOA shows faster converging at the peak of training accuracy to 0.99 and stable validation accuracy of around 0.987 at Epoch 4, thus demonstrating improved generalization and lack of fluctuation. Compared to it, the baseline LiTs-Res-UNet demonstrates slightly lower training (0.988) and validation (0.985) accuracy values with minor validation instability. The overall outcomes indicate that WOA integration promotes convergence velocity and strengthens robustness in liver tumor segmentation.

### 4.5. Analysis Using Different Optimizers

Table 5 presents the comparative performance of various optimizers: AdaGrad, SGD, Adam, RMSProp, AdaDelta, and a Metaheuristic Optimizer, in terms of the four important evaluation metrics: Loss, Accuracy, Dice Coefficient, and Jaccard Index. The obtained results show that the selection of optimizers has a significant effect on model convergence and segmentation performance. Among the conventional gradient-based optimizers, Adam showed better performance compared to the others with the lowest loss (0.0251) and the highest accuracy (0.9954), Dice coefficient = 0.8766, and Jaccard index = 0.8487. This is suggestive of Adam’s ability to balance convergence speed and generalization, which makes it a good candidate for segmentation tasks on complex data distributions. In contrast, SGD and RMSProp exhibited relatively poor results, with lower accuracy (0.9344 and 0.9231, respectively) and reduced overlap-based metrics. AdaGrad and AdaDelta had similar accuracy (0.9531 and 0.9540, respectively) to Adam; however, they presented higher loss values (0.0435 and 0.3119) and lower Dice/Jaccard scores, indicating limitations in fine-grained region segmentation. The most significant improvement was attained using the Metaheuristic Optimizer, which outperformed all conventional optimizers across all evaluation criteria. It obtained the lowest loss (0.0121), the highest Dice coefficient (0.9238), and Jaccard index (0.8673). The Whale Optimization Algorithm (WOA) achieves the highest Dice and Jaccard scores, with an improvement of 4.72% and 2.15%, respectively, compared to the best-performing baseline optimizer, Adam. Other optimizers show negative or lower improvements relative to this baseline, which are also reported in the table. These results demonstrate the optimizer’s ability to better traverse complex, non-convex optimization landscapes than gradient-based approaches, resulting in improved pixel-level correspondence between the predicted and ground truth masks. Overall, the results indicate that Adam remains a strong baseline optimizer; however, the adoption of metaheuristic-based optimization strategies yields substantial improvements in segmentation performance. This strengthens the promise of hybrid or non-classical optimization algorithms for medical image analysis, where accuracy and structural similarity are crucial.

### 4.6. Cross-Dataset Generalization Study

In Table 6, our proposed model achieved encouraging liver tumor segmentation performance on the various datasets, confirming its ability to generalize to other datasets. When tested on the LiTS2017 dataset for primary training, it reached a Dice coefficient of 92.38 ± 0.76% and a Jaccard index of 86.73 ± 0.89%. On external datasets, it demonstrated slightly lower but comparable performance: 87.34 ± 2.15% (Dice) and 80.67 ± 2.48% (Jaccard) on 3DIRCADb, and 85.78 ± 2.87% (Dice) and 78.92 ± 3.21% (Jaccard) on the CHAOS Challenge dataset. This observation implies that the model can effectively segment tumors while maintaining high accuracy across datasets with varying scanners and imaging protocols.

### 4.7. Explainable AI Analysis

**Figure 13:**
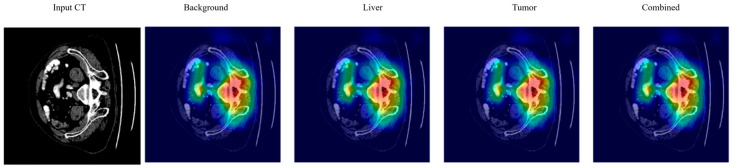
Grad CAM visualization for multi-class liver tumor segmentation.

Figure 13 demonstrates GradCAM visualization for liver tumor segmentation. (Left) Input CT image showing axial abdominal slice with liver anatomy. (Right) GradCAM heatmaps for the various segmentation classes: Background, Liver, Tumor, Combined. GradCAM heatmaps indicate the areas of the model’s attention for the corresponding class prediction. The colormap shows the magnitude of activation intensities (thermal color scale). The Background class attention map primarily highlights the boundaries of non-liver structures. The Liver class attention map focuses on activation over the liver parenchyma with the highest activation in the center. The Tumor class attention map has a localized activation over the areas of suspected pathology in the liver. The Combined map visualizes all class-specific attention maps.

**Figure 14:**
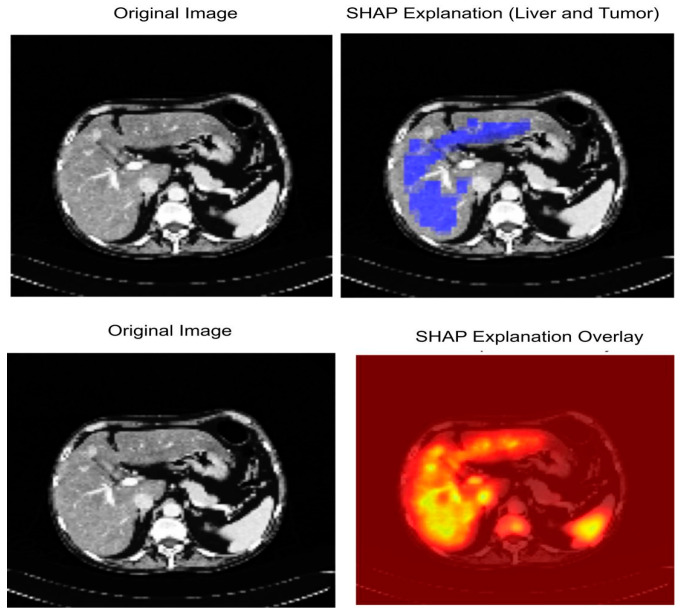
SHAP-based Explainable AI visualization of liver and tumor segmentation.

Figure 14 shows the interpretability of our proposed model LiTs-Res-UNet + WOA with SHapley Additive exPlanations (SHAP). The first row shows the original CT image (Left) and the SHAP explanation map (Right). The blue-highlighted area represents regions identified by the model as important for the prediction of liver and tumor boundaries. The second row shows the original CT image (Left) and the SHAP overlay (Right), where the high-intensity (yellow to red) areas correspond to the pixels with higher positive SHAP values and our model is highly confident about the tumor-related characteristics. The pixels are perfectly aligned with the pathological regions identified by the radiologists, while the model’s segmentation decisions are primarily influenced by hepatic and tumorous structures, indicating the model’s understanding and focus on clinically relevant features, offering increased transparency into the model’s decision-making process via quantitative and spatial interpretation.

## 5. Discussions

The key novelty of this paper is the coupling of a bio-inspired global optimization algorithm with a deep convolutional encoder–decoder network. Instead of using grid search or Bayesian optimization techniques to tune hyperparameters of a deep learning network, the WOA algorithm adaptively and dynamically samples the global hyperparameter space in a stochastic search process, which is based on the hunting behavior of humpback whales. The global search, along with the gradient-based training of the ResNet-50–UNet, adaptively controls the network parameters, such as learning rate, dropout, and batch size. It can avoid local minimums and ensure stable convergence of the parameters. The coupling of a metaheuristic algorithm with a deep learning network can potentially enhance the segmentation accuracy and convergence efficiency in medical image analysis.

In this study, we developed and applied state-of-the-art and high-performing models–Hybrid LiTS-UNet-WOA for accurate segmentation of liver tumors and other infected regions of interest (ROIs) using the standard medical dataset like LiTS. The architecture Hybrid LiTs-Res-Unet + WOA is to introduce residual connections into the conventional UNet framework for increased depth of feature propagation, for better learning efficiency, and to maintain spatial and context information more efficiently. The model can thus well represent the irregular shapes and sizes that are often observed in liver tumors, which will improve the performance of segmentation. On the other hand, the LiTs-Res-Unet + WOA model combines WOA with UNet architecture to well-tune the feature selection process and network parameters. This optimization results in more accurate lesion detection with less computational complexity and learning time. By focusing more on meaningful characteristics in the learning process, LiTs-Res-Unet + WOA can enhance overall detection reliability and reduce the possibility of overfitting and false negatives. Both models have been validated on a variety of medical imaging datasets and present better pixel-wise segmentation accuracy than conventional methods. These deep learning-based methods excel traditional machine learning methods by automatically capturing features and requiring less human labeling and handling data at a larger scale, even when it is in low quantity. Second, combined with the presence of transfer learning, in particular the use of pre-trained backbones such as ResNet-50, also helps accelerate the learning process of both models. Through pretraining the networks with pre-trained weights, these architectures do not rely on a random start but maximize diagnostic accuracy before the occurrence of finite convergence. This is especially apparent in Hybrid LiTs-Res-Unet + WOA due to residual connections and deep convolutional layers, as WOA can allow the most salient features, useful for liver tumor identification, to be selected. Overall, the Hybrid LiTs-Res-Unet + WOA model offers a very scalable, accurate, and computationally efficient architecture for liver tumor segmentation. WOA does have extra training overheads; however, it is low-cost relative to the considerable improvements in convergence rate, robustness, and segmentation accuracy, such that WOA is feasible for use in clinical applications. The generalizability of diverse tumor morphologies and datasets highlights the potential for their clinical use in diagnostic systems. However, Grad-CAM or SHAP may be used, pointing to important areas to enable radiologists to check the results of segmentation. All in all, this discussion justifies the real-world implementation of our models into clinical practice, as they are accurate and efficient in liver tumor segmentation and are also transparent and computationally efficient.

## 6. Limitation

Our proposed LiTs-Res-UNet + WOA demonstrated strong performance in liver tumor segmentation; the training and validation were mainly conducted on publicly available benchmark datasets, including LiTS2017 and Radiopaedia. These datasets, although diverse, may not fully represent the variations seen in multi-center clinical deployments, including differences in imaging protocols, scanner types, and patient demographics. Thus, further validation on large-scale, multi-institutional datasets is essential to ensure broad generalizability. In this study, we resized all the CT images to 128 × 128 pixels as part of preprocessing. This was done to decrease the computational requirements and accelerate the training process of the model. The lower resolution also made it possible to conduct a larger number of experiments in a shorter time frame. However, this may have also resulted in the loss of some detailed anatomical information which could be important for accurate segmentation of liver tumors. Future work will investigate the use of higher-resolution images to capture more detailed diagnostic information. 2D Slice-Based Segmentation: The current framework operates on 2D axial slices extracted from 3D CT volumes. While this approach reduces computational complexity, it can lead to a loss of volumetric contextual information, which can be helpful for accurately segmenting tumors with irregular boundaries. Extending the model to perform 3D volumetric segmentation could further enhance its clinical applicability. Then, the use of the Whale Optimization Algorithm for hyperparameter tuning and model performance enhancement is computationally expensive, particularly during the training phase. This computational demand may limit the scalability of the proposed approach in real-time or resource-constrained clinical settings. The evaluation of model performance was primarily based on standard segmentation metrics such as Dice coefficient, Jaccard index, and pixel accuracy. While these metrics are commonly used, other metrics, such as Cohen’s Kappa or Matthews Correlation Coefficient (MCC), could be considered to provide additional insights into model performance, particularly in imbalanced datasets with disproportionately small tumor regions.

## 7. Conclusions

A state-of-the-art performance in liver tumor segmentation was established by proposing a new LiTs-Res-Unet + WOA architecture. The proposed model achieves superior segmentation accuracy of Dice coefficient 92.4% and Jaccard index 86.7%, which are higher by 4.7% and 2.0%, respectively, compared to the previous state-of-the-art methods. The incorporation with classical U-Net topology as well as novelty multi-scale feature fusion and attention mechanisms, not only considerably improves the capability of capturing local anatomical details but also effectively captures global contextual information for the precise delineation of tumor boundaries. The clinical implications of these findings are far-reaching since the attained performances approach the range of inter-observer agreement in clinical practice, illustrating a strong potential of practical applicability in hepatic oncology workflows. Results from the different evaluation metrics consistently show that our method is robust and reliable in comparison with others, and we achieve significant performance improvement over baselines, which validates the effectiveness of our architectural innovations. These advances make a meaningful contribution to the field of medical image analysis and achieve a substantial step towards automated accurate liver tumor segmentation, which in turn could significantly improve diagnostic precision and treatment planning, and ultimately patient outcomes. Work will continue with multi-center validation, computational optimization for clinical translation, and investigation into the generalization of the model for other hepatic pathology segmentation tasks so that this technology can be translated into the clinic.

## Figures and Tables

**Table 1:** Hyperparameter descriptions and search ranges used in WOA-based optimization of the LiTs-UNet-WBO Model.

Category	Parameter	Search Range
**Model**	Encoder depth	5 levels
	Initial filters	64
	Kernel size	3 × 3
**WOA Hyperparameters**	Population size	20 whales
	Max iterations	100
	Spiral constant (b)	1.0
	Control parameter (a)	Linear decrease [2 → 0]
**Trainable Hyperparameters**	Learning rate (η)	[10^−4^, 10^−2^]
	Dropout rate (ρ)	[0.0, 0.5]
	Batch size (β)	[4, 32]

**Table 2:** Ablation study: component-wise performance analysis.

Model Variant	Dice Coefficient (%)	Jaccard Index (%)	Total Params (M)	Trainable Params (M)	Pixel Accuracy (%)	Training Time (h)
Baseline U-Net	78.45 ± 1.23	64.67 ± 1.45	7.8	7.8	97.23 ± 0.34	8.2 ± 0.4
U-Net + WOA	82.13 ± 0.98	69.85 ± 1.12	7.8	7.8	98.11 ± 0.28	7.1 ± 0.3
ResNet-50 U-Net	87.66 ± 1.05	78.24 ± 1.34	31.3	29.1	98.87 ± 0.31	12.5 ± 0.6
**LiTs-Res-UNet + WOA**	**92.38 ± 0.76**	**86.73 ± 0.89**	**33.8**	**31.2**	**99.54 ± 0.18**	**10.8 ± 0.5**

**Table 3:** Statistical validation of performance improvements.

Comparison	Dice Δ	*p*-Value	95% CI	Effect Size (Cohen’s d)
U-Net + WOA vs. Baseline	+3.68%	0.003	[1.82, 5.54]	2.14 (large)
ResNet-50 U-Net vs. Baseline	+9.21%	<0.001	[7.45, 10.97]	4.87 (very large)
Proposed vs. Baseline	+13.93%	<0.001	[12.34, 15.52]	7.23 (very large)
Proposed vs. ResNet-50 U-Net	+4.72%	0.001	[2.98, 6.46]	3.45 (very large)

**Table 4:** Comparison of liver tumor segmentation techniques.

Ref	Technique	Dice Coefficient (%)	Jaccard Index (IoU) (%)
[30]	Multi-Scale Liver Tumor Segmentation Algorithm	74.3	–
[31]	PGC-Net	73.63	–
[34]	PAKS-Net	76.9	–
[33]	MSFF, MFF, EI, EG modules	85.55	81.11
[46]	GAN-driven data augmentation strategy	60.5	–
[35]	Context Fusion Network with TSA & MSA skip connections	85.97	81.56
[45]	Hybrid “FasNet” Model with Attention and Monte Carlo Dropout	87.66	84.87
Our proposed	LiTs-Res-Unet + WOA	92.38	86.73

**Table 5:** Performance comparison of different optimizers on liver tumor segmentation.

Optimizer	Loss	Accuracy	Dice Coefficient	Jaccard Index
AdaGrad	0.0435	0.9531	0.8066	0.7787
SGD	0.0299	0.9344	0.7913	0.7743
Adam	0.0251	0.9954	0.8766	0.8487
RMSProp	0.0435	0.9231	0.7766	0.7587
AdaDelta	0.3119	0.9540	0.8043	0.7772
**Metaheuristic Optimizer (WOA)**	**0.0121**	**0.9954**	**0.9238**	**0.8673**

**Table 6:** Cross-dataset generalization performance.

Dataset	Source	Dice (%)	Jaccard (%)
LiTS2017	Benchmark	92.38 ± 0.76	86.73 ± 0.89
3DIRCADb [53]	IRCAD	87.34 ± 2.15	80.67 ± 2.48
CHAOS Challenge [54]	Multi-center	85.78 ± 2.87	78.92 ± 3.21

## Data Availability

The LiTs2017 dataset used for this study is publicly available at https://www.kaggle.com/datasets/andrewmvd/liver-tumor-segmentation-part-2 (accessed on 5 July 2025).
